# Supplementary material for: Minor sequence modifications in temporin B cause drastic changes in antibacterial potency and selectivity by fundamentally altering membrane activity
Source: Sci Rep. 2019 Feb 4;9:1385. doi: 10.1038/s41598-018-37630-3 (PMC6362004; doi:10.1038/s41598-018-37630-3)
Supplement: Supplementary file 1 — Supplementary Data 1 [file 41598_2018_37630_MOESM1_ESM.pdf]

Minor sequence modifications in temporin B cause drastic changes in antibacterial potency and selectivity by fundamentally altering membrane activity

Giorgia Manzo[1], Philip M. Ferguson[1], V. Benjamin Gustilo[1], Charlotte Hind[2], Melanie Clifford[2], Tam T. Bui[3], Alex. F. Drake[3], R. Andrew Atkinson[3], Mark J. Sutton[2], Giovanna Batoni[4] Christian D. Lorenz[5], David A. Phoenix[6], A. James Mason[1*]

[1]Institute of Pharmaceutical Science, School of Cancer & Pharmaceutical Science, King's College London, Franklin-Wilkins Building, 150 Stamford Street, London, SE1 9NH, United Kingdom

[2]Technology Development Group, National Infection Service, Public Health England, Salisbury, UK

[3]Centre for Biomolecular Spectroscopy and Randall Division of Cell and Molecular Biophysics, King's College London, New Hunt's House, London SE1 1UL, United Kingdom

[4]Department of Translational Research and New Technologies in Medicine and Surgery, University of Pisa, Pisa, Italy

[5]Department of Physics, King's College London, London WC2R 2LS, United Kingdom

[6]School of Applied Science, London South Bank University, 103 Borough Road, London SE1 0AA, United Kingdom

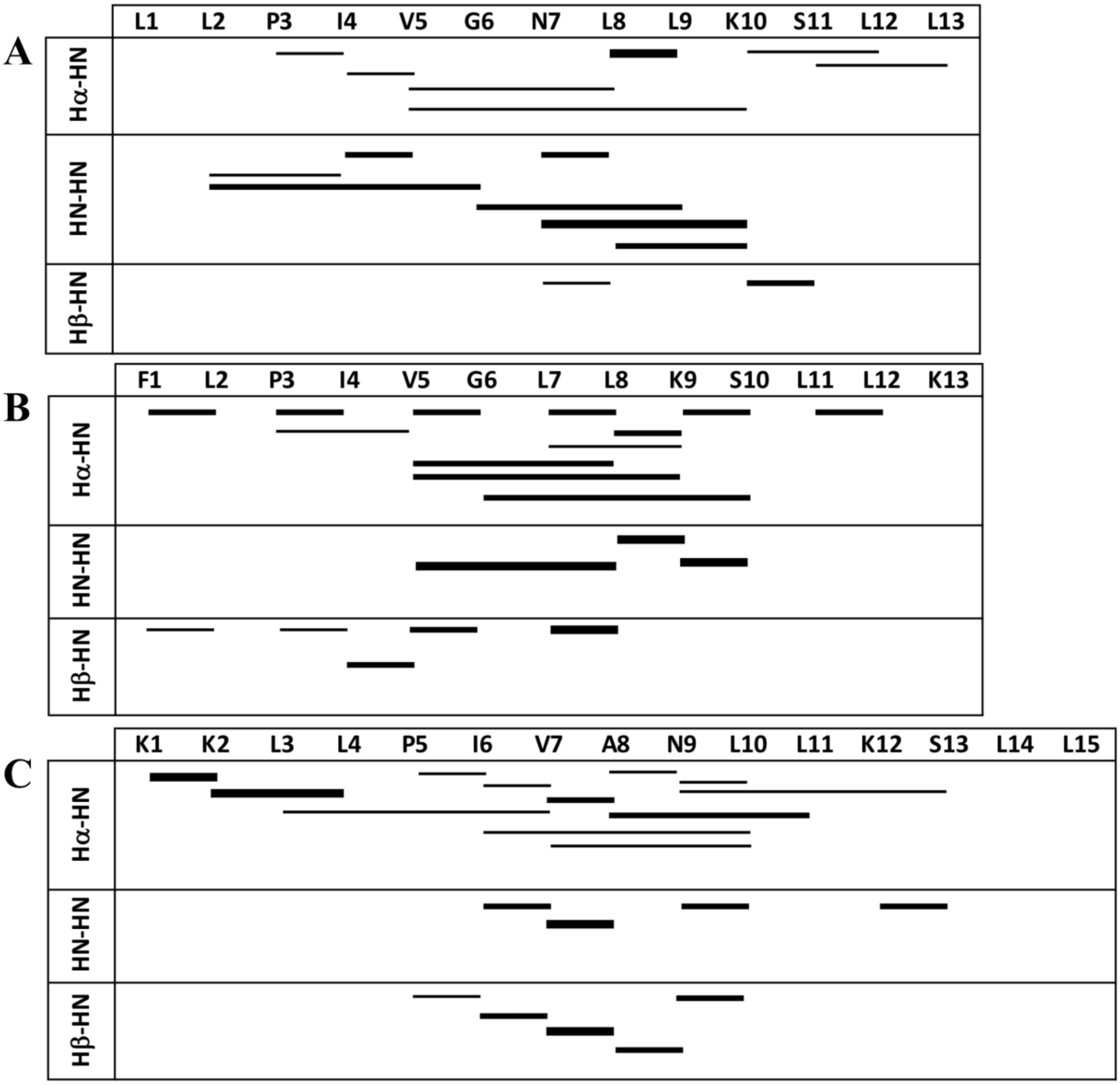

Figure S1. Interproton dipolar through-space interactions (NOEs) for temporin B (A), temporin B L1FK (B) and temporin B KKG6A (C). The interactions are reported as lines connecting the two residues of the sequence. Lines thickness are proportional to the relative intensity of the cross-peak in the NOESY spectrum (categorised strong, medium and weak)

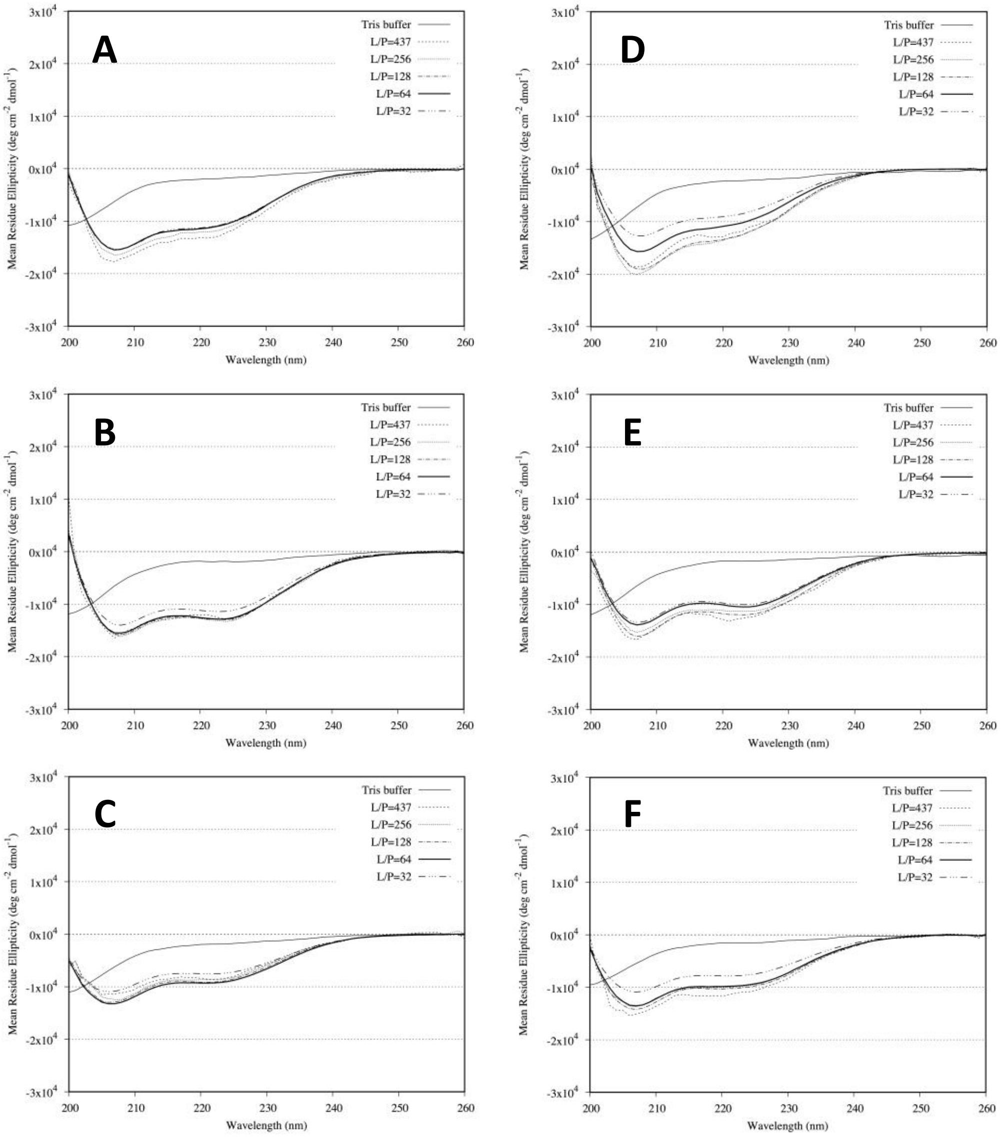

Figure S2. Dose dependent variations in secondary structure in model membranes. Far-UV CD spectra of temporin B (A/D), temporin B L1FK (B/E) and temporin B KKG6A (C/F) were acquired in the presence of 5 mM POPE/POPG (75/25) (A-C) or POPG (D-F) SUVs, 5 mM Tris-amine buffer, pH 7.00 and 100 mM NaCl.

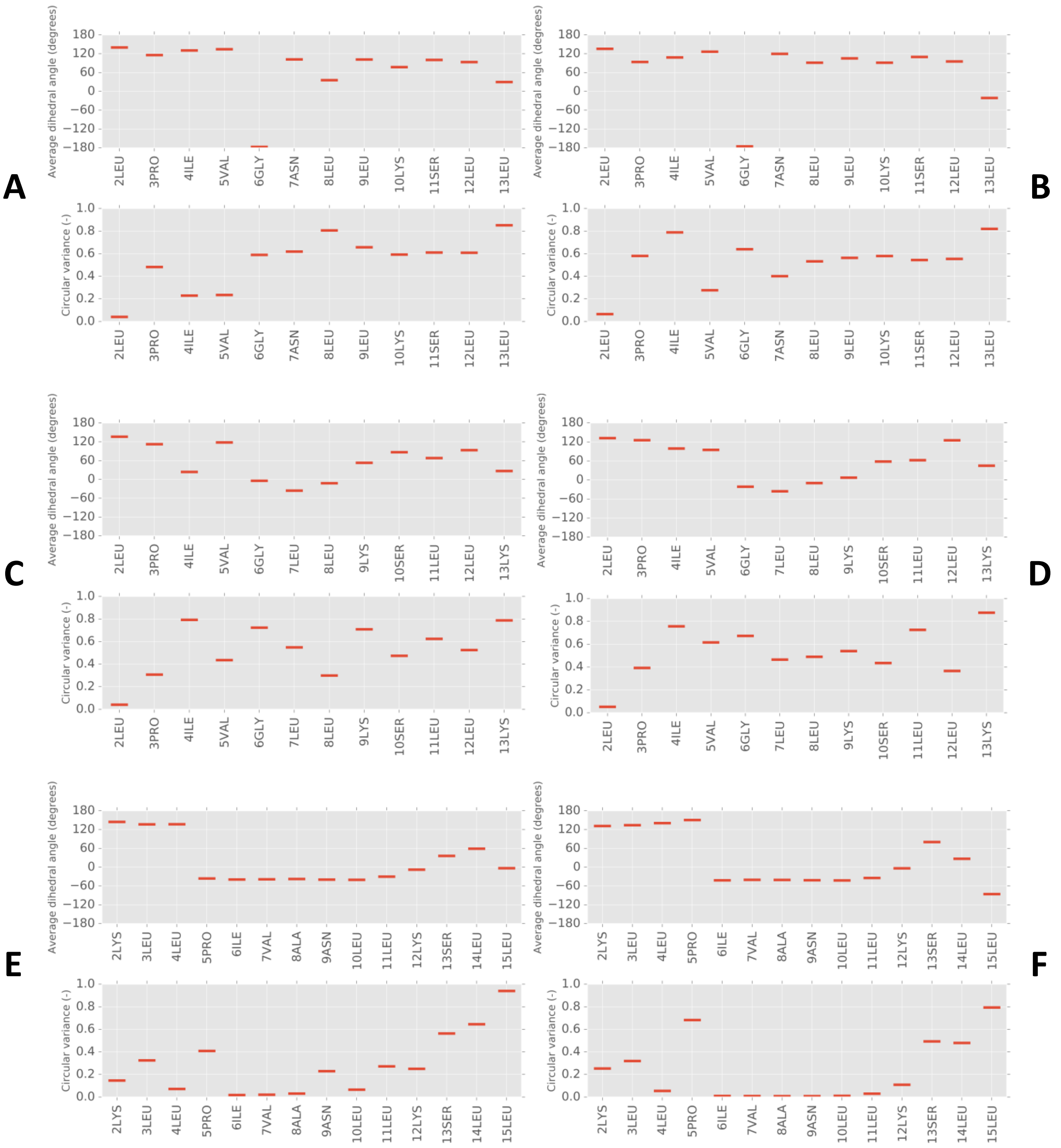

Figure S3. Secondary structure analysis of temporin B peptides from MD simulations in model membranes (duplicate runs). Dihedral angles (psi) and their circular variance are shown for each residue averaged over 100 ns of simulation and eight peptides in temporin B (A/B), temporin B L1FK (C/D) and temporin B KKG6A (E/F) peptides when binding to POPE/POPG (A/C/E) or POPG (B/D/F) membranes. Data shown are replicates for that shown in Fig. 2 to show consistency across repeated 100 ns simulations.

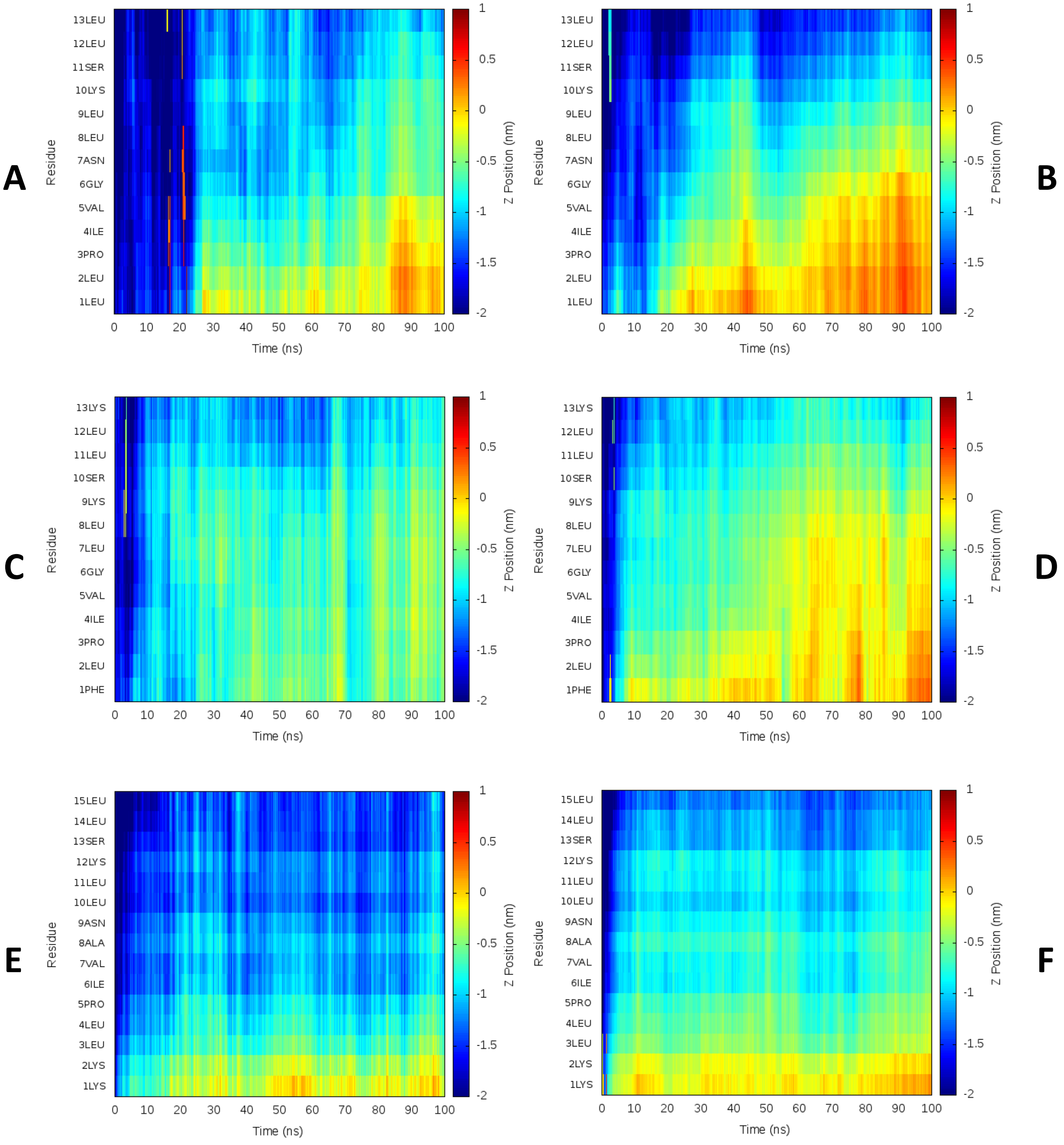

Figure S4. Temporin B peptide topology during 100 ns MD simulation in model membranes (duplicate runs). The depth of temporin B (A/B), temporin B L1FK (C/D) and temporin B KKG6A (E/F) insertion is shown as the Z-position relative to the phosphate group for each residue averaged over all eight peptides in each simulation. Positive or negative values indicate the peptides are below or above the phosphate group, respectively for peptides in POPE/POPG (A/C/E) or POPG (B/D/F). Data shown are replicates for that shown in Fig. 3 to show consistency across repeated 100 ns simulations.

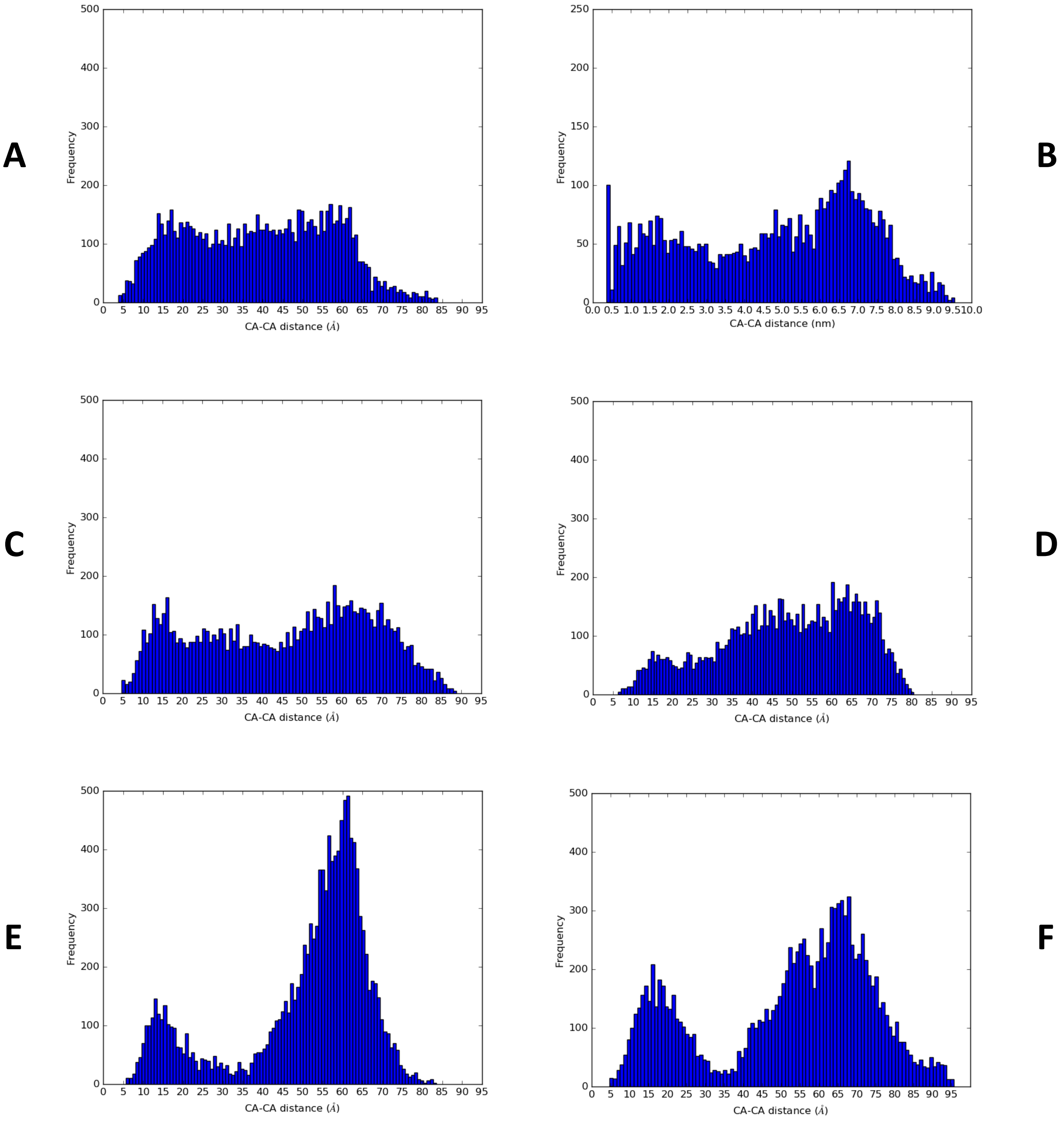

Figure S5. Temporin B peptides aggregation during 100 ns MD simulation in model membranes (duplicate runs). The probability of temporin B (A/B), temporin B L1FK (C/D) and temporin B KKG6A (E/F) aggregation is reported as frequency histogram of the Cα-Cα distances, respectively for peptides in POPE/POPG (A/C/E) or POPG (B/D/F) membranes. Data shown are replicates for that shown in Fig. 4 to show consistency across repeated 100 ns simulations.

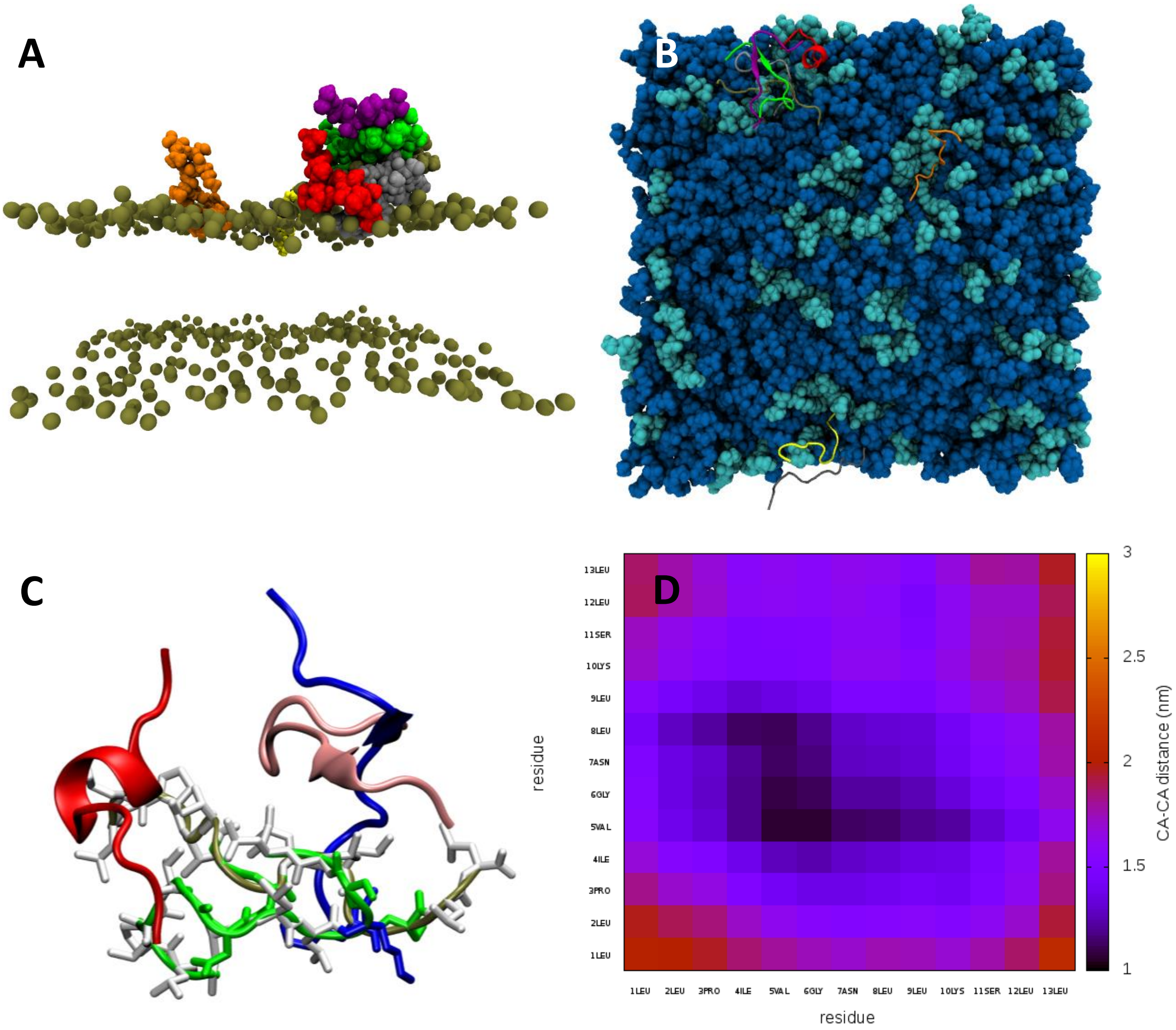

Figure S6. Interaction of temporin B with POPE/POPG bilayer. The depth of peptides insertion is shown in the side view snapshot (A). The top view snapshot shows the tendency to aggregate for the eight peptides (B). (C) shows an example of peptides aggregating as a tetramer. Basic, polar, acidic and hydrophobic residues are coloured in blue, green, red and white respectively. The heatmap (D) shows the stronger interactions between the residues as average Cα-Cα.

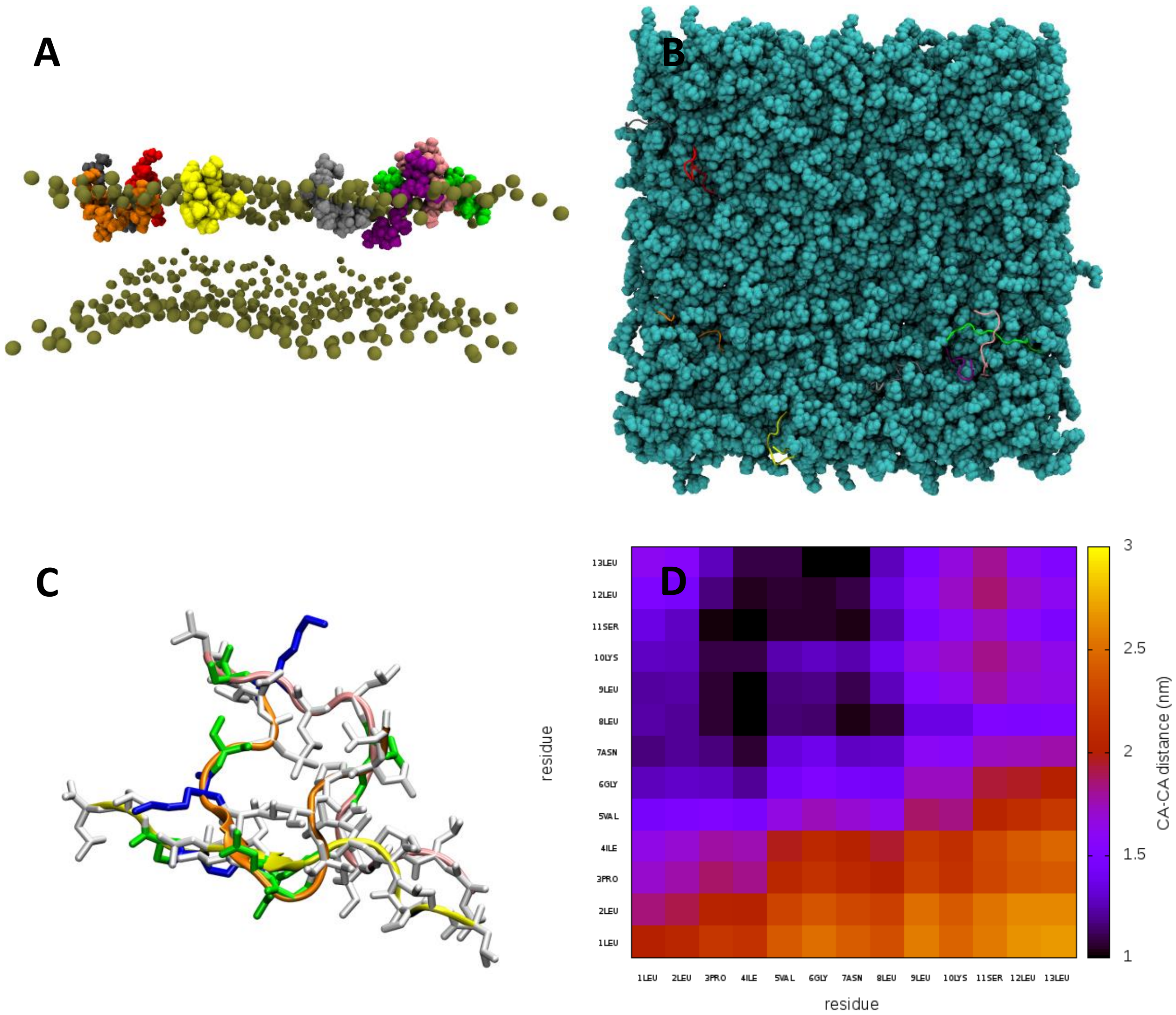

Figure S7. Interaction of temporin B with POPG bilayer. The depth of peptides insertion is shown in the side view snapshot (A). The top view snapshot shows the tendency to aggregate for the eight peptides (B). (C) shows an example of peptides aggregating as a trimer. Basic, polar, acidic and hydrophobic residues are coloured in blue, green, red and white respectively. The heatmap (D) shows the stronger interactions between the residues as average Cα-Cα.

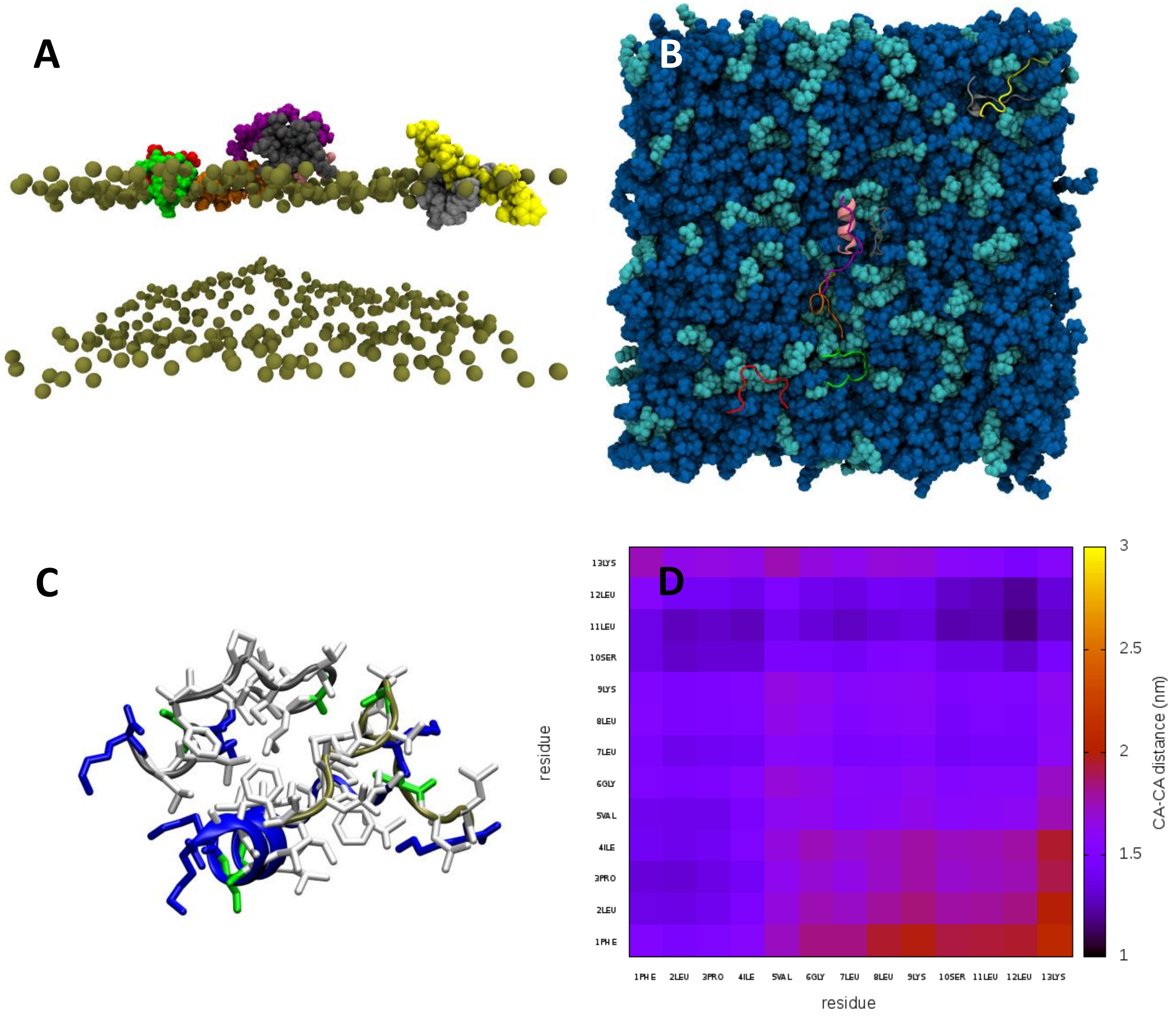

Figure S8. Interaction of temporin B L1FK with POPE/POPG bilayer. The depth of peptides insertion is shown in the side view snapshot (A). The top view snapshot shows the tendency to aggregate for the eight peptides (B). (C) shows an example of peptides aggregating as a trimer. Basic, polar, acidic and hydrophobic residues are coloured in blue, green, red and white respectively. The heatmap (D) shows the stronger interactions between the residues as average Cα-Cα.

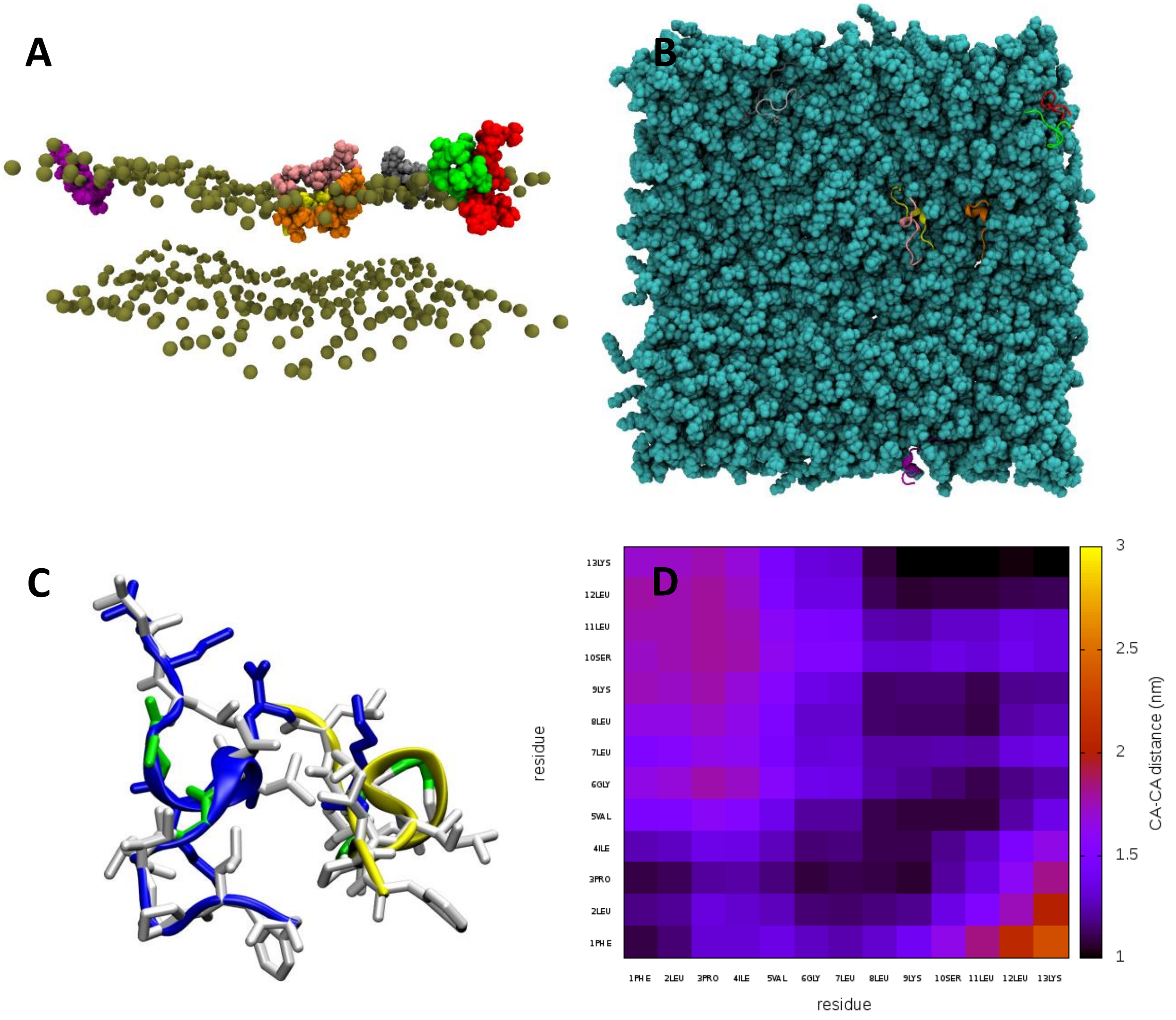

Figure S9. Interaction of temporin B L1FK with POPG bilayer. The depth of peptides insertion is shown in the side view snapshot (A). The top view snapshot shows the tendency to aggregate for the eight peptides (B). (C) shows an example of peptides aggregating as a dimer. Basic, polar, acidic and hydrophobic residues are coloured in blue, green, red and white respectively. The heatmap (D) shows the stronger interactions between the residues as average Cα-Cα.

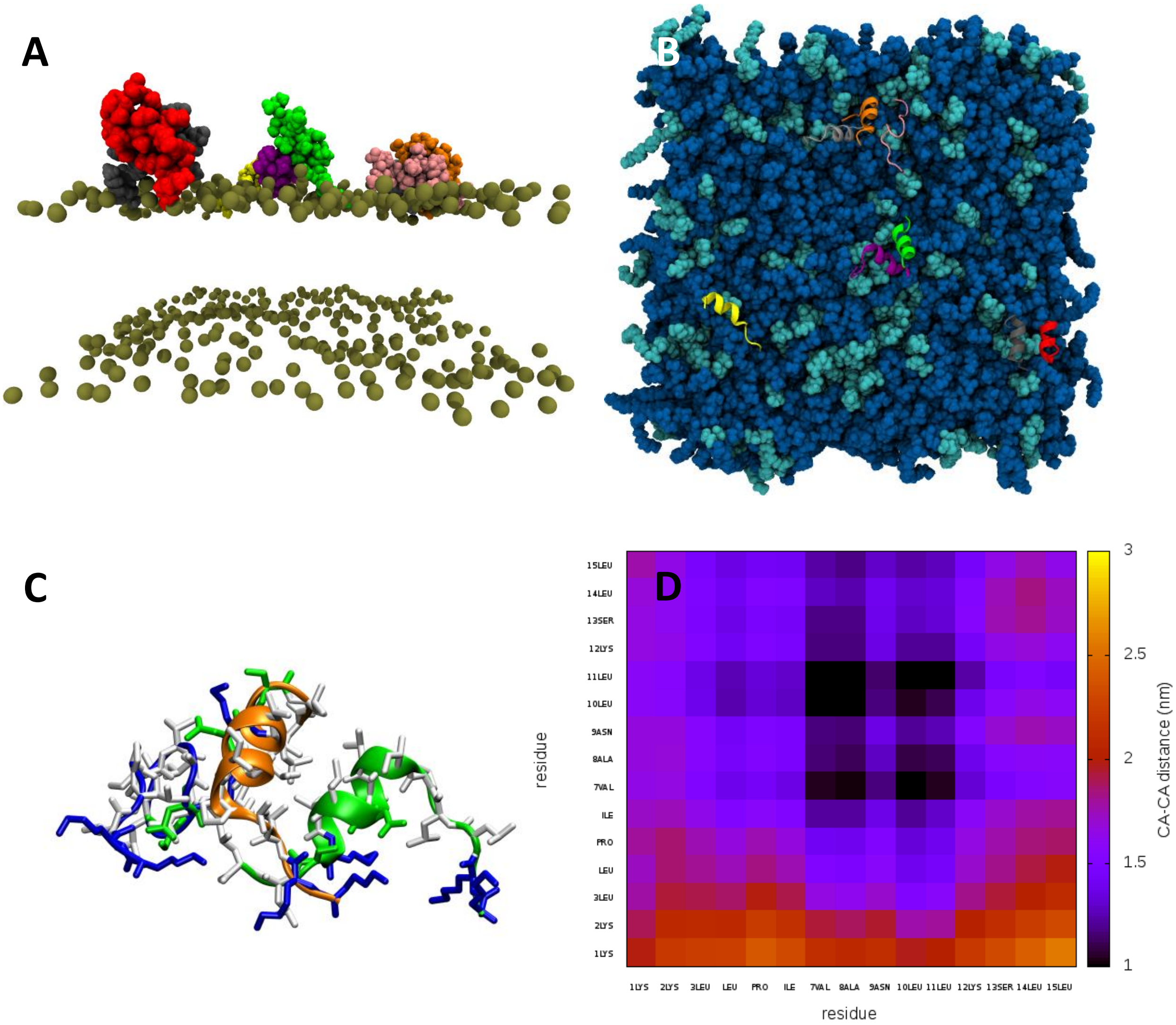

Figure S10. Interaction of temporin B KKG6A with POPE/POPG bilayer. The depth of peptides insertion is shown in the side view snapshot (A). The top view snapshot shows the tendency to aggregate for the eight peptides (B). (C) shows an example of peptides aggregating as a trimer. Basic, polar, acidic and hydrophobic residues are coloured in blue, green, red and white respectively. The heatmap (D) shows the stronger interactions between the residues as average Cα-Cα.

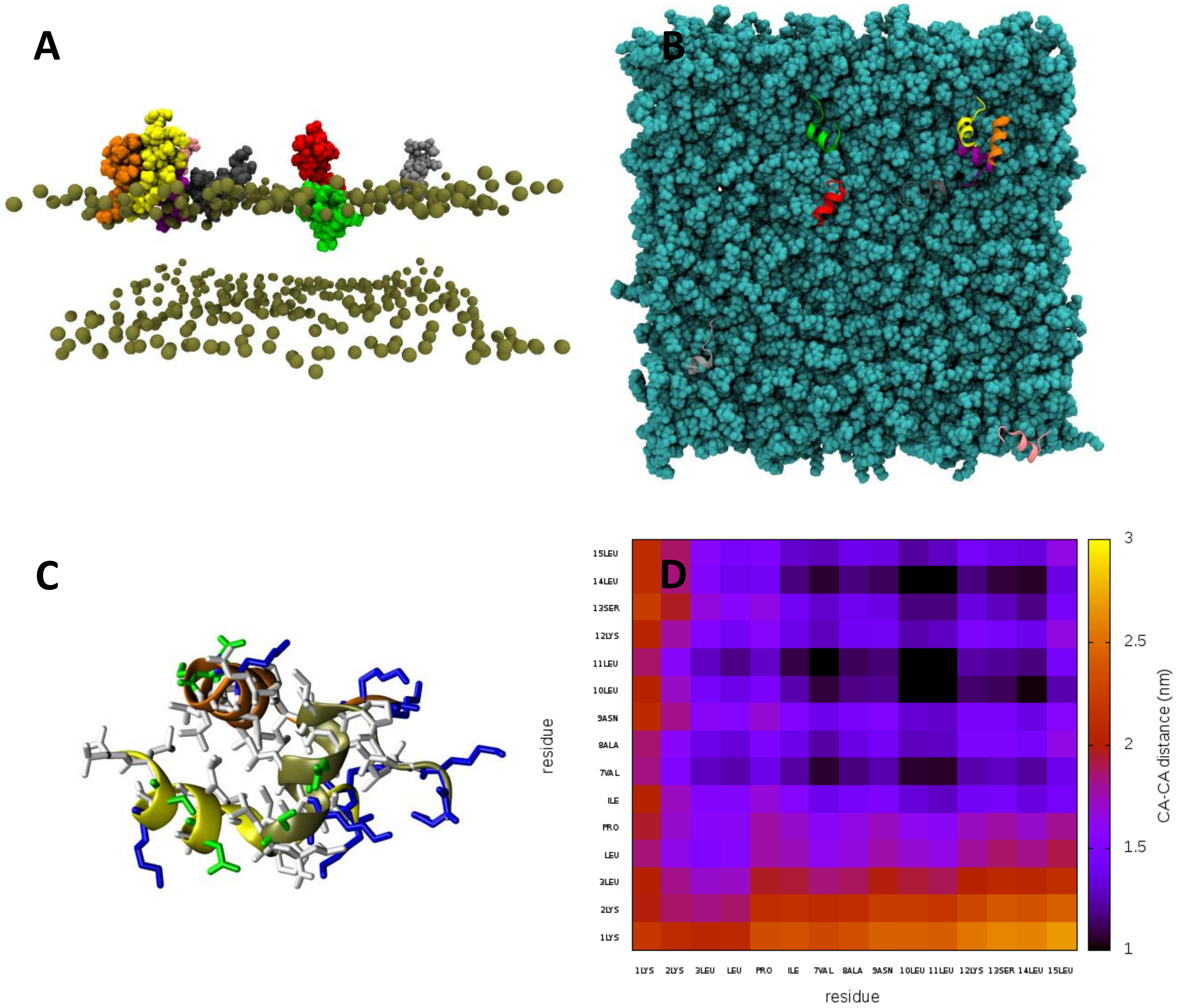

Figure S11. Interaction of temporin B KKG6A with POPG bilayer. The depth of peptides insertion is shown in the side view snapshot (A). The top view snapshot shows the tendency to aggregate for the eight peptides (B). (C) shows an example of peptides aggregating as a trimer. Basic, polar, acidic and hydrophobic residues are coloured in blue, green, red and white respectively. The heatmap (D) shows the stronger interactions between the residues as average Cα-Cα.

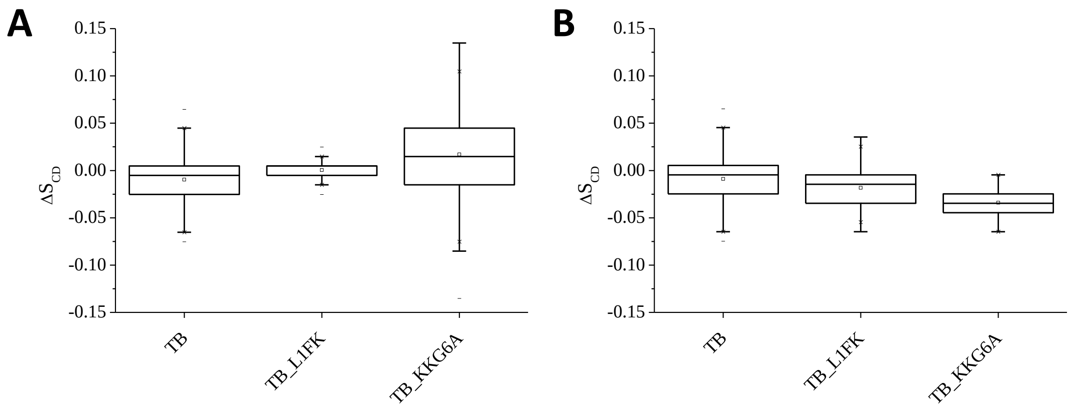

Figure S12. Disordering of annular lipids during MD simulations of temporin B peptides in POPE/POPG bilayers. Disordering of POPG (A) or POPE (B) lipids located within 4 Å of any of the eight peptides of the indicated temporin B peptide. Results are shown for the last 50 ns of a 100 ns simulations containing eight peptide molecules, 384 zwitterionic POPE lipids and 128 anionic POPG lipids. The data from MD simulations qualitatively reproduces those obtained for model membranes in the steady-state (Fig. 5B/C).

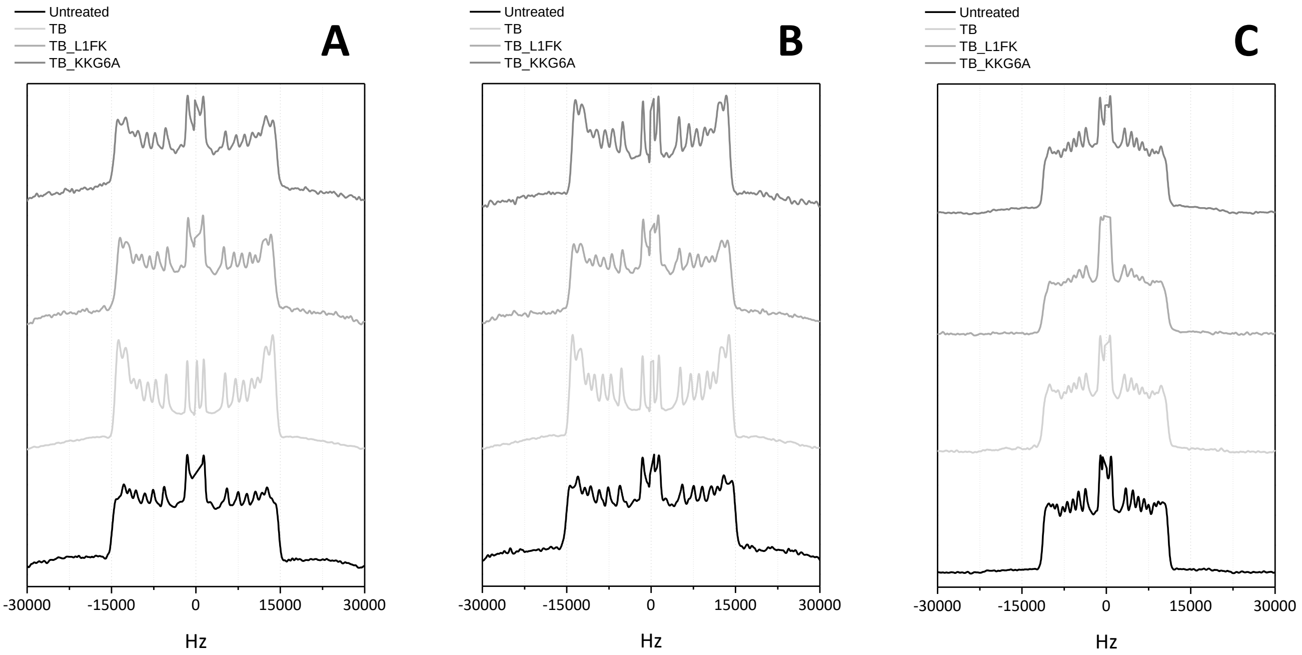

Figure S13. ^{2}H echo NMR spectra of multilamellar vesicles challenged with 2 mol% of each temporin B peptide. MLVs **were** composed of POPE/POPG-d31 (75:25) (A), POPE/POPE-d31/POPG (50:25:25) (B) or POPG/POPG-d31 (75:25) (D) in 5 mM Tris-amine buffer, pH 7.00 and 100 mM NaCl.
